# Modulating Glial Activity with Focused Ultrasound against Alzheimer's disease

**DOI:** 10.1002/alz70855_103265

**Published:** 2025-12-23

**Authors:** Sophie Morse

**Affiliations:** ^1^ Imperial Hosptial, London, United Kingdom

## Abstract

**Background:**

Ultrasound is an ideal therapeutic technology to modulate cellular activity as it is non‐invasive, targeted and can reach any region of the brain. In this study, we developed focused ultrasound to modulate the activity of the innate immune cells of the brain, microglia, to reduce inflammatory responses which accelerate disease progression.

**Method:**

After measuring the levels of proinflammatory cytokines (TNF‐α, IL‐1β and IL‐6) released by LPS‐inflamed microglia in vitro, we tested such levels in the brain of LPS‐inflamed mice receiving a single whole‐brain ultrasound treatment with varying ultrasound parameters. In a 5xFAD Alzheimer's mouse model, we are now testing whether chronic treatments, once a week for 5 days, lead to microglial changes as well as synaptic and behavioural outcomes.

**Result:**

We found that using low frequency (500 kHz) and low pressures (0.2 MPa) of ultrasound for just 5 minutes can significantly reduce proinflammatory cytokines (TNF‐α, IL‐1β and IL‐6) induced by microglia in vitro. This effect lasts for up to 72 hours post‐treatment. Critically, for the first time, we showed that ultrasound can reverse microglial phenotype and release anti‐inflammatory cytokines (IL‐10, IL‐4) which are essential for tissue repair and brain homeostasis. In vivo, a single ultrasound treatment was also able to significantly reduce pro‐inflammatory and increase anti‐inflammatory cytokine levels in the brain.

**Conclusion:**

This work indicates that focused ultrasound could be used as a powerful technology to modulate the activity of microglia, reducing proinflammatory cytokine expression, with the potential to modulate other unwanted cellular glial behaviours in the brain to treat neurodegenerative diseases.

